# Secondary cutaneous Epstein-Barr virus-associated diffuse large B-cell lymphoma in a patient with angioimmunoblastic T-cell lymphoma: a case report and review of literature

**DOI:** 10.1186/1746-1596-7-7

**Published:** 2012-01-19

**Authors:** Qing-Xu Yang, Xiao-Juan Pei, Xiao-Ying Tian, Yang Li, Zhi Li

**Affiliations:** 1 Department of Pathology, Huizhou Municipal Central Hospital, 41, Eling Road North, Huizhou 516001, China; 2 Department of Pathology, The First Affiliated Hospital, Sun Yat-sen University, 58, Zhongshan Road II, Guangzhou 510080, China; 3 School of Chinese Medicine, Hong Kong Baptist University, 7, Baptist University Road, Kowloon Tong, Hong Kong, China

**Keywords:** Angioimmunoblastic T-cell lymphoma, Epstein-Barr virus, secondary B-cell lymphoma, skin lesion

## Abstract

Only a few cases of extranodal Epstein-Barr virus (EBV)-associated B-cell lymphomas arising from patients with angioimmunoblastic T-cell lymphoma (AITL) have been described. We report a case of AITL of which secondary cutaneous EBV-associated diffuse large B-cell lymphoma (DLBCL) developed after the initial diagnosis of AITL. A 65-year-old Chinese male patient was diagnosed as AITL based on typical histological and immunohistochemical characteristics in biopsy of the enlarged right inguinal lymph nodes. The patient initially received 6 cycles of chemotherapy with CHOP regimen (cyclophosphamide, vincristine, adriamycin, prednisone), but his symptoms did not disappear. Nineteen months after initial diagnosis of AITL, the patient was hospitalized again because of multiple plaques and nodules on the skin. The skin biopsy was performed, but this time the tumor was composed of large, polymorphous population of lymphocytes with CD20 and CD79a positive on immunohistochemical staining. The tumor cells were strong positive for EBER by in situ hybridization. The findings of skin biopsy were compatible with EBV-associated DLBCL. CHOP-R chemotherapy (cyclophosphamide, doxorubicin, vincristine, prednisone and rituximab) was then administered, resulting in partial response of the disease with pancytopenia and suppression of cellular immunity. To our knowledge, this is the first case of cutaneous EBV-associated DLBCL originated from AITL in Chinese pepole. We suggest the patients with AITL should perform lymph node and skin biopsies regularly in the course of the disease to detect the progression of secondary lymphomas.

**Virtual slides:**

The virtual slide(s) for this article can be found here:

http://www.diagnosticpathology.diagnomx.eu/vs/1197421158639299

## Background

Angioimmunoblastic T-cell lymphoma (AITL) is one of the most common subtypes of peripheral T-cell lymphomas, accounting for approximately 15-20% of all cases, or 1-2% of all non-Hodgkin lymphomas [1]. AITL is frequently accompanied by a polymorphic nature including fever, skin eruptions, polyclonal hypergammaglobulinemia, generalized lymphadenopathy and hepatosplenomegaly [2]. Histopathologically, the primary site of AITL is lymph node characterized by the effacement of the lymph node architecture, the increase of arborizing high endothelial venules and the infiltration of clear small- to medium-sized lymphocytes, along with the increase of B-immunoblasts that usually contain Epstein-Barr virus (EBV). AITL patients exhibit immunodeficiency secondary to the neoplastic process. Expansion of EBV-positive B cell is thought to be a consequence of underlying immune dysfunction [3]. In rare condition, secondary EBV-associated B cell lymphoma may occur together with AITL. However, to our knowledge, so far only 22 cases of AITL developed EBV-associated secondary B cell lymphomas have been described in English literature [4-14]. Herein, we report a case of secondary EBV-associated diffuse large B cell lymphoma occurring in the skin of a patient with lymph node AITL.

## Case presentation

A 65-year-old Chinese male patient presented with a 6-month history of enlarged right inguinal lymph nodes without pain and fever and initially sought examination and treatment at our hospital in September, 2009. Physical examination revealed multiple enlarged lymph nodes easily identified in the right inguinal region, and the spleen was palpable 2 cm below the left costal margin without discrete masses or tenderness. Maculopapular eruptions could be found in the trunk. There was no remarkable edema and masses observed in the skin. The laboratory results including blood count, differential, liver and renal function were within the normal range. Eosinophilia and hypergammaglobulinemia were not observed. Computed tomography (CT) scan disclosed the enlargement of right inguinal and visceral lymph nodes, as well as hepatosplenomegaly. Test for EBV exhibited both viral capsid antigen (VCA) and EBV-associated antigen positive.

**Figure 1:**
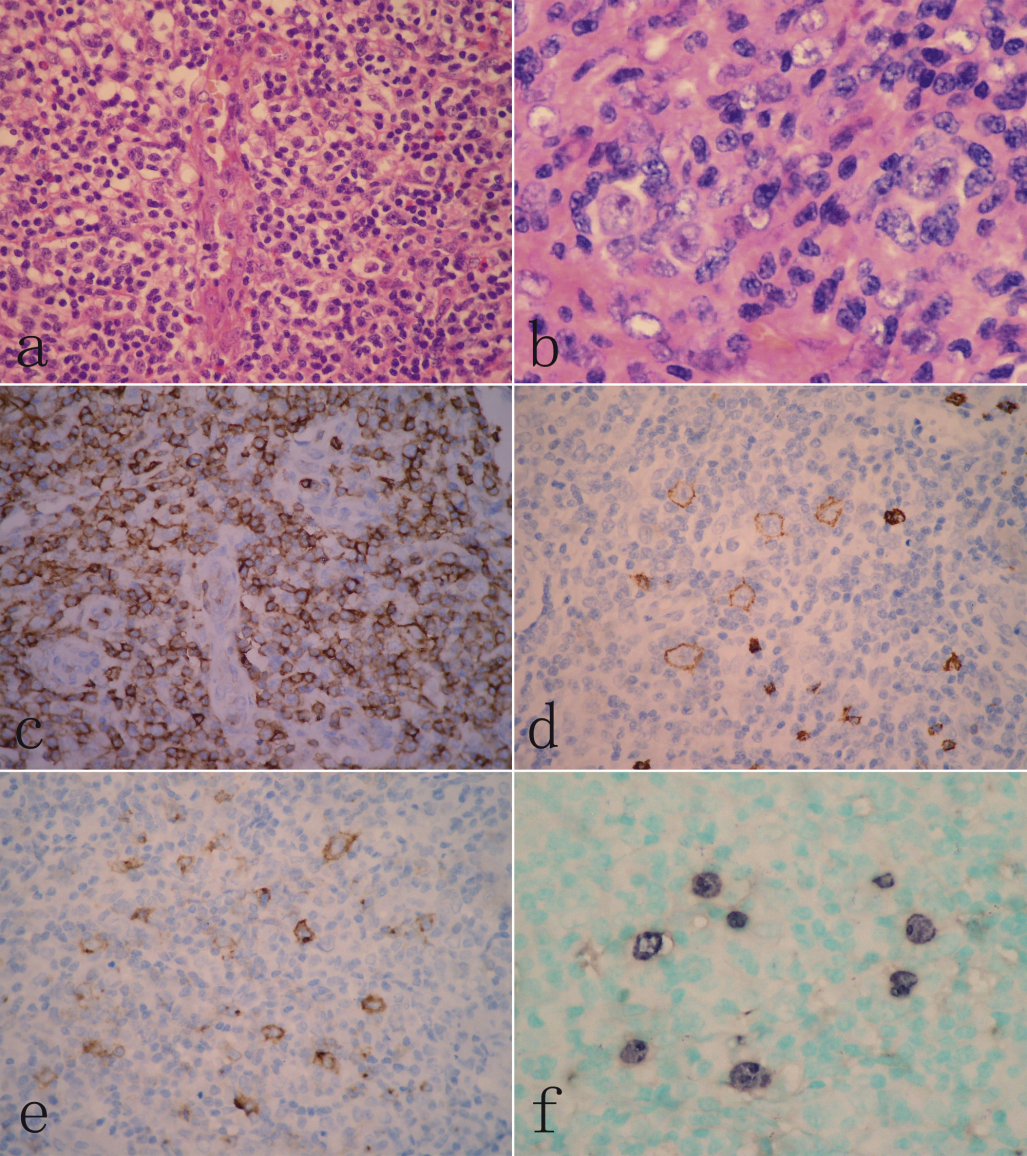
**Histopathological findings of lymph node at the initial presentation**. (A) The architecture of lymph node was effaced by a diffuse polymorphic infiltration composed of small- to medium-sized lymphoid cells, immunoblastic cells and scattered eosinophils around high-endothelial venules. (B) Multinucleated cells with eosinophilic nucleoli resembling Reed-Stemberg (RS) cells could be observed in the lymph node. (C) Immunohistochemical examination revealed that infiltrated small to medium-sized lymphoid cells were diffusely positive for CD3, but large immunoblast-like cells and RS-like cells were positive for CD20 (D) and CD30 (E).These larger cells were also positive for EBER by in situ hybridization (F).(A, H&E staining, with original magnification ×400; B, H&E staining, with original magnification ×600; C-E, immunohistochemical staining, with original magnification ×400; F, EBER-in situ hybridization, with original magnification ×400).

The patient then underwent biopsy of right inguinal lymph node, but the biopsy of skin lesion was not performed at that time. The biopsy tissue was routinely processed and stained by hematoxylin and eosin (H&E). Under the microscopy, the normal architecture of lymph node was destroyed by polymorphic cellular infiltration composed of diffused small to medium-sized lymphoid cells along with plasma cells and eosinophils. The infiltrated lymphocytes had clear cytoplasm with mildly irregular nuclei. Some of large immunoblast-like lymphoid cells with large distinct nuclei and clear cytoplasm were observed to intermingle with those infiltrated lymphocytes. Numerous arborizing high-endothelial venules were prominent in the background of lymph node. In addition, scattered Reed-Sternberg (RS)-like cells with irregular multilobated nuclei and large eosinophilic nucleoli were also present in the node (Figure 1A-B). Immunohistochemically, the infiltrated small to medium-sized lymphoid cells were strongly positive for CD3, CD45RO, CD10 and CD4, but negative for CD20, CD79a, PAX-5, CD56, MUM-1 and CD30. The large immunoblast-like cells and scattered RS-like cells showed immunoreactive for CD20, CD79a and CD30. The proliferation of follicular dendritic cells highlighted by CD21 and CD23 was prominent throughout node, and entrapped the high-endothelial venules. By in situ hybridization assay, EBER-positive signal was observed in either scattered large B immunoblasts or RS-like cells (Figure 1C-F). Based on clinical presentation and histological findings, a histological diagnosis of angioimmunoblastic T-cell lymphoma (AITL) was made according to the criteria of WHO classification [3].

After the diagnosis of AITL, the patient received 6 cycles of chemotherapy with CHOP regimen (cyclophosphamide, vincristine, adriamycin, and prednisone), but his symptoms did not disappear. The patient was followed up without any treatment for nearly one year after completing the whole chemotherapy courses. There was no further complaint from the patient during the follow-up period. In April, 2011, nineteen months after initial diagnosis the patient was readmitted to our hospital due to presentation of multiple plaques and nodules on the skin. Skin examination showed irregular plaques and nodules ranging from 0.5 to 2.5 cm in diameters in the trunk and extremities. CT scan revealed multiple enlargements of right inguinal and visceral lymph nodes as well as hepatosplenomegaly. Bone marrow aspiration was normal. The skin biopsies were performed in the skin lesion located at the left upper arm.

**Figure 2:**
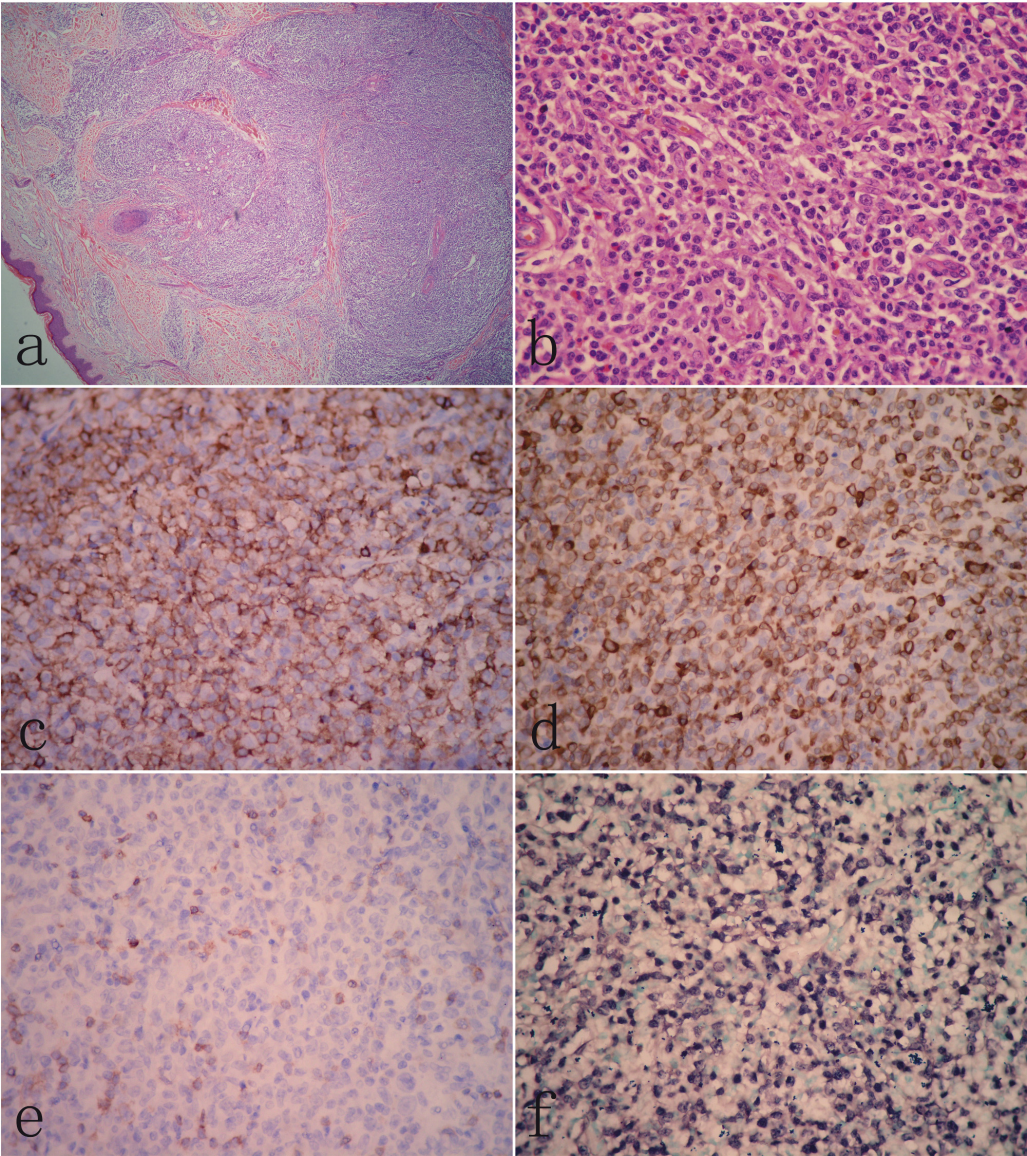
**Histological findings of skin lesion**. (A) Low power view of skin lesion showed diffuse infiltration of lymphoid cells in dermal and subcutaneous tissue without epidermotropism. (B) Large atypical lymphoid cells with prominent nucleoli were observed in the skin lesion. Atypical large lymphoid cells were stained positively with CD20 (C) and CD79a (D). However, CD3 positive cells in skin lesion were small lymphocytes with scattered distribution (E). (F) Most of atypical large cells were positive for EBER by in situ hybridization. (A, H&E staining, with original magnification ×40; B, H&E staining, with original magnification ×400; C-E, immunohistochemical staining, with original magnification ×400; F, EBER-in situ hybridization, with original magnification ×400).

Microscopically, dermal and subcutaneous tissue was infiltrated by sheets of a monotonous population of medium- to large-sized atypical lymphoid cells without epidermotropism. Cellular atypia exhibited irregular nuclei, large nucleoli and vesicular chromatin with a brisk mitotic rate, atypical mitotic figures (Figure 2A-B). Immunohistochemically, the atypical cells were strongly positive for CD20, CD79a and Bcl-6, but negative for CD3, CD5, and CD45RO. EBV detection by in situ hybridization for EBERs showed diffuse positive reaction (Figure 2C-F). These findings were compatible with diffuse large B cell lymphoma (DLBCL). Sections of the lymph node biopsy performed one year earlier were reviewed. The structure and cytologic features of skin lesion were distinct from those of the lymph node. There was a diffuse infiltrate of predominantly small to medium-sized lymphocytes with unambiguous T cell markers by immunohistochemical analysis in lymph node. These tumor cells were negative reaction to EBER assay. However, the infiltrated large sized neoplastic cells in the skin lesion were identified as B lymphocytes and positive to EBER assay. Based on these findings, a histological diagnosis of AITL-developed secondary EBV-associated DLBCL of skin was made.

After diagnosis, the patient received a cycle of R-CHOP regimen (rituximab, cyclophosphamide, doxorubicin, vincristine, and prednisone) and achieved a partial response. However, pancytopenia and suppression of cellular immunity were observed. The patient refused further treatment of R-hyperCVAD regimen (rituximab, cyclophosphamide, vincristine, doxorubicin, and dexamethasone) and was discharged from hospital. The further following up was not performed due to contact loss of the patient.

Angioimmunoblastic T-cell lymphoma (AITL) was previously regarded to be an atypical reactive process. In 1974, Frizzera *et al*. firstly termed it as "angioimmunoblastic lymphadenopathy with dysproteinemia (AILD)" [15], of which AILD was described as a reactive lymphoproliferative disorder of T lymphocytes [16]. AITL was considered as a malignant transformation of AILD, but the histological distinguishing AITL from AILD was quite difficult. In the past, the presence of clusters of cells with abundant clear cytoplasm was regarded as one of the most important morphological criteria and used to distinguish AITL from AILD. However, subsequent molecular and cytogenetic studies revealed that clonal TCR gene rearrangement and chromosomal abnormalities were identified in the majority of AILD [17,18], which indicated most cases previously diagnosed as AILD actually represented AITL. In 2001, WHO classification of tumor of hematopoietic and lymphoid tissue suggested AITL was most likely to arise as a peripheral T-cell lymphoma. As both terms of AITL and AILD were in common use, they could be used interchangeably in clinics. However, some researchers argued that a few cases of AILD with atypical or oligoclonal proliferation might precede the development of AITL, and should be classified as a distinct entity of preneoplastic process [19,20]. Although debate remained, we considered AILD might represent the existence of stages in the development of AITL rather than independent disease entities.

Although the pathogenesis of AITL remains unclear, it is currently thought to most likely derived from germinal-center T-helper cells (T_FH_) [21]. The primary site of AITL is lymph node and virtually all patients present with generalized lymphadenopathy. Histological examination of the lymph nodes exhibits nearly complete effacement of the follicular architecture, a mixed lymphoid infiltrate, and numerous high endothelial venules in an expanded T-cell zone. In some cases, the lymph nodes show diffuse obliteration of their architecture by lymphoid infiltration consisting of lymphocytes, immunoblasts, plasma cells, and histiocytes, together with numerous high endothelial venules surrounded by an expanded network of follicular dendritic cells. Immunohistochemically, the neoplastic cells express most pan T-cell antigens such as CD2, CD3 and CD5. The majority of the proliferating cells are CD4 positive or, less often, CD8 positive [3]. Attygalle *et al*. demonstrated that CD10 was a phenotypic marker, which specifically identified the tumor cells of 90% of AITL [22]. Dorfman *et al*. reported that programmed death-1 (PD-1), a member of the CD28 costimulatory receptor family, is expressed by germinal center-associated T cells in AITL [23]. De Leval *et al*. indicated tumor cells in AITL could overexpress CXCL13, which is the characteristic of normal T_FH _cells [21]. These researchers suggest those immunophenotypes are helpful to distinguish AITL from atypical paracortical hyperplasia and other peripheral T-cell lymphomas as well as diagnosing extranodal dissemination. In our case, not only histological examination of lymph node demonstrated a morphological characteristic of AITL, but also the presence of T-cell linage with aberrant CD4 and CD10 expression identified in neoplastic cells by immunohistochemical staining which also strongly supported a diagnosis of AITL at initial lymph node biopsy.

It is well known that most cases of AITL are frequently complicated with EBV infection, but the neoplastic T cells are EBV negative. Almost all cells infected by EBV are B-cells showing normal histological findings [3,24]. However, in rare condition, the complication of EBV-associated B cell lymphoma in AITL has been reported. The immune dysregulation present in AITL may have permitted EBV infection or reactivation, which could play a role in the proliferation of B-cell immunoblasts and clonal expansion of an immortalized EBV-infected B-cell clone. Therefore, the development of B-cell lymphoma can be a consequence of the disease progression of AITL [3,6]. To the best of our knowledge, so far only 22 cases of AITL-developed secondary EBV-associated B cell lymphoma have been described in the literature (Table 1) [4-14]. Similar to our case, most of previously reported cases showed only AITL on initial biopsy, and showed B-cell lymphoma (with or without coexistent AITL) on follow-up biopsy. The time interval between two tumors ranged from 5 to 96 months. AITL with simultaneous B-cell lymphoma or primary B-cell lymphoma in the background of AITL could be found in 6 cases. "Composite AITL and B-cell lymphoma" was suggested by Xu *et al*. when the simultaneous appearance of these two distinct lymphomas developed in the same anatomic site [6]. It was commonly considered that secondary EBV-associated B-cell lymphomas in AITL raised from EBV-infected B cells in lymph node, and we found indeed that EBV could be detected in initial AITL in most of previous cases. However, EBV-infected B-cell was not detected in the 6 cases before the development of secondary B-cell lymphoma by EBER-in situ hybridization method. Whether or not specific mechanism involved in the sequential development of EBV-associated B-cell lymphoma in AITL derived from non-EBV-infected cells should be investigated further. Seven cases have been reported that the secondary B-cell lymphoma develop in extranodal organs, including skin, bone marrow, duodenal bulbous, lung, ileum and soft tissue [4,7-12], and only two cases as well as ours presented skin involvement by secondary B-cell lymphoma [4,8]. The present case is the first report on cutaneous sequential development of EBV-associated DLBCL from AITL in Chinese patients. Although the prognosis of AITL once complicated by EBV-associated DLBCL is not well known, extranodal involvement of lymphomas is a poorer sign. We found in previous AITL cases, the patients with extranodal involvement by B-cell lymphoma presented shorter survival time than those with secondary B-cell lymphoma in lymph nodes. Based on these data, it is thought the present case might have an aggressive biological behavior and a less favorable prognosis.

**Table 1:** Clinicopathological features of patients with AITL-developed secondary EBV-associated B cell lymphoma described in present and previous reports

**caseNo**.	Authors (yr.)	Age/sex	Tumor site		Interval (mo)	Histotype of BCL*	Treatment		Clinical Outcome
							
			AITL	BCL			AITL	BCL	
1	Abruzzo LV (1993) [4]	46/M	LN	SK	26	Large-cell immunoblastic type	Cyclophosphamide	N/D	Died of multiorgan failure
2	Park S (2002) [5]	55/F	LN	LN	48	BCL	CHOP	CHOP-R	CR and alive 12 mo later
3	Xu Y (2002) [6]	48/F	LN	LN	0	DLBCL	CHOP and ESHAP		CR and relapsed a few months later
4	Zettl A (2002) [7]	68/M	LN	ST	34	DLBCL	Observation	Glucocorticoids	Died 4 mo later of tuberculosis
5		47/M	LN	LN	29	DLBCL	CC; BMT	CC; involved field radiation	Died 3 mo later of Aspergillus pneumonia
6		61/M	LN	LN	96	Plasmacytoma	N/Av	N/Av	Alive with disease 24 mo later
7	Hawley RC (2006) [8]	69/F	LN	SK	56	DLBCL	CHOP	R-hyperCVAD	Died 54 mo later
8	Attygalle AD (2007) [9]	28/M	LN	BM	24	DLBCL	CHOP	Gemcitabine	No response and died 3 mo later
9		60/F	LN	LN	8	DLBCL	CHOP	CHOP-Et	No response and died 3 mo later
10		59/M	LN	LN	8	DLBCL	N/Av	CC	PR and alive with disease 68 mo later
11		72/M	LN	LN	84	DLBCL	N/Av	N/Av	N/A
12		78/F	LN	LN	0	DLBCL	Thalidomide		PR
13	Willenbrock K (2007) [10]	76/F	LN	LN	8	DLBCL	N/Av	N/Av	N/Av
14		46/F	LN	BM	5	DLBCL	N/Av	N/Av	N/Av
15		84/M	LN	LN	0	DLBCL	N/Av	N/Av	N/Av
16		72/M	LN	LN	0	DLBCL	N/Av	N/Av	N/Av
17		65/M	LN	LN	0	CD30-positive BCL	N/Av	N/Av	N/Av
18		60/F	LN	LN	0	DLBCL	N/Av	N/Av	N/Av
19	Weisel KC (2008) [11]	59/M	LN	DB and lung	11	DLBCL	Fudarabine+ CHOP	N/D	Died 2 weeks later
20	Takahashi T (2010) [12]	66/F	LN	Ileum	24	DLBCL	THPCOP CHASE	N/D	Died 1 mo later of respiratory failure
21	Skugor ND (2010) [13]	36/F	LN	LN	11	DLBCL	FED	CHOP-R and stem cell transplantation	CR
22	Huang J (2011) [14]	64/M	LN	LN	47	DLBCL	IHOP	CHOP-R	Alive with disease 13 mo later
23	Present case	65/M	LN	SK	19	DLBCL	CHOP	CHOP-R	PR

*, all B-cell lymphoma with EBV positive; AITL, angioimmunoblastic T-cell lymphoma; BCL, B-cell lymphoma; DLBCL, diffuse large B-cell lymphoma; LN, lymph node; SK, skin; ST, soft tissue; BM, bone marrow; DB, Duodenal bulbus; Interval 0, AITL with simultaneous B-cell lymphoma or primary B-cell lymphoma in the background of AITL; BMT, bone marrow transplantation; N/Av, not available; N/D, not done; PR, partial response; CR, complete remission; CHOP, cyclophosphamide, vincristine, adriamycin, and prednisone; ESHAP, etoposide, methyl prednisolone, cytarabine (ara-C) and cisplatin; hyperCVAD, cyclophosphamide, vincristine, doxorubicin, and dexamethasone; IHOP, ifosfamide, doxorubicin, vincristine, and prednisone; THPCOP, cyclophosphamide, pirarubicin, vincristine, prednisolone; CHASE, cyclophosphamide, cytarabine, etoposide, dexamethasone; FED, fludarabine, cyclophosphamide, dexamethasone; Et, etoposide; R, rituximab; CC, combination chemotherapy, details not available;

The presence of large immunoblast cells and scattered RS-like cells in AITL might create diagnostic confusion with other neoplastic condition of lymphoid tissue. The main differential diagnoses include classical Hodgkin lymphoma (cHL) and T cell/histiocyte-rich large B-cell lymphoma (THRLBCL). THRLBCL is characterized by a limited number of scattered, large, atypical B cells embedded in a background of abundant T cells and frequently histiocytes. Immunohistochemically, large atypical cells express pan B-cell markers and no expression of CD30, and are lack of EBV infection. The background of THRLBCL is composed of variable number of CD68-positive histiocytes and CD3-, CD5-positive small T cells rather than CD4-, CD10- and CXCL13-positive small to medium-sized T_FH _cells. Numerous high endothelial venules surrounded by an expanded network of follicular dendritic cells highlighted by CD21 and CD23 will be useful to distinguish AITL form THRLBCL. In this present case, an interesting finding was the presence of occasional CD30 and EBV-positive RS-like cells within inflammatory background of lymph node. This histological finding was similar to the morphological feature of cHL. However, recognition of cytologic atypia or immunophenotypic aberrancy in the background T cells is critical for making the differential diagnosis between AITL and cHL.

In conclusion, secondary B-cell lymphoma in AITL is rare. Herein we report the first case of cutaneous EBV-associated DLBCL sequential to AITL in Chinese patients. Extranodal involvement by secondary lymphoma in the patients with AITL might imply a poor prognosis. Therefore, clinicians should keep in mind that there is a possibility of sequential development of B-cell lymphoma after AITL. It is necessary to perform lymph node and skin biopsies regularly to detect the progression to secondary lymphomas for patients with AITL.

## Consent

Written informed consent was obtained from the patient for publication of this case report and any accompanying images. A copy of the written consent is available for review by the Editor-in-Chief of this journal.

## Competing interests

The authors declare that they have no competing interests.

## Authors' contributions

Q-XY and X-JP made contributions to acquisition of clinical data, and analysis of the histological features by H&E staining. X-YT drafted the manuscript. ZL revised manuscript critically for important intellectual content and had given final approval of the version to be published. YL carried out the immunoassays. All authors read and approved the final manuscript.
